# Supplementary material for: Experimental Investigation of Mode Localization’s Bifurcation Topology Transfer in Electrostatically Coupled Tuning Fork Resonators
Source: Sensors (Basel). 2024 Feb 28;24(5):1563. doi: 10.3390/s24051563 (PMC10934075; doi:10.3390/s24051563)
Supplement: Supplementary file 1 [file sensors-24-01563-s001.zip › sensors-2800966-supplementary.pdf]

Derivation of the mathematical model for feedthrough in Electrostatic Coupled Tuning Fork Resonators

Ming Lyu[1,2*], Xiang Zhi[1,2], Na Yan[1,2], Rongjian Sun[3]

According to the principle of parallel plate capacitor action, the motion current is generated due to the change in displacement of the movable pole plate, and the motion current outputs of the detecting electrode 1 and detecting electrode 2 can be deduced as follows:

$$\begin{aligned} i_1(\tilde{t}) &= \int_0^l \left(V_{\mathrm{dc1}} \frac{dCs}{dt} H_2 \right) d\tilde{x} = \int_0^l \left(V_{\mathrm{dc1}} \frac{dCs}{d\tilde{w}_1} \frac{d\tilde{w}_1}{d\tilde{t}} H_2 \right) d\tilde{x} \\ &= \int_0^1 \left(\frac{V_{\mathrm{dc1}} \varepsilon_0 b_1 \phi_1(\tilde{x}) \dot{q}_1(\tilde{t}) H_2}{(\mathrm{g_a} - \tilde{w}_1)^2} \right) d\tilde{x} \approx \frac{V_{\mathrm{dc1}} \varepsilon_0 b_1}{\mathrm{g_a}^2} \int_0^l \phi_1(\tilde{x}) H_2 d\tilde{x} \dot{q}_1(\tilde{t}) = \eta_1 \dot{q}_1(\tilde{t}) \end{aligned} \tag{1}$$

$$\begin{aligned} i_2(\tilde{t}) &= \int_0^l \left(V_{\mathrm{dc2}} \frac{dCs}{dt} H_2 \right) d\tilde{x} = \int_0^l \left(V_{\mathrm{dc2}} \frac{dCs}{d\tilde{w}_2} \frac{d\tilde{w}_2}{d\tilde{t}} H_2 \right) d\tilde{x} \\ &= \int_0^1 \left(\frac{V_{\mathrm{dc2}} \varepsilon_0 b_1 \phi_2(\tilde{x}) \dot{q}_2(\tilde{t}) H_2}{(\mathrm{g_a} + \tilde{w}_2)^2} \right) d\tilde{x} \approx \frac{V_{\mathrm{dc2}} \varepsilon_0 b_1}{\mathrm{g_a}^2} \int_0^l \phi_2(\tilde{x}) H_2 d\tilde{x} \dot{q}_2(\tilde{t}) = \eta_2 \dot{q}_2(\tilde{t}) \end{aligned} \tag{2}$$

where η_1 and η_2 are electromechanical conversion factors to describe the amount of capacitance change per unit displacement:

$$\eta_1 = \frac{V_{\mathrm{dc1}} \varepsilon_0 b_1}{\mathrm{g_a^2}} \int_0^l \phi_1(\tilde{x}) H_2 d\tilde{x}, \quad \eta_2 = \frac{V_{\mathrm{dc2}} \varepsilon_0 b_1}{\mathrm{g_a^2}} \int_0^l \phi_2(\tilde{x}) H_2 d\tilde{x} \tag{3}$$

According to equations (1) and (2), the dynamic currents i_1 and i_2 are proportional to the product of the velocity terms $\dot{q}_1(\tilde{t})$ 、 $\dot{q}_2(\tilde{t})$ and the electromechanical conversion coefficients η_1 and η_2, and thus the linear simplified equations can be written as:

$$L_{11} \frac{di_1(t)}{dt} + R_{11} i_1(t) + \frac{1}{C_{11}} \int i_1(t) dt + \mathrm{C}_c \int i_2(t) dt = F_1 V_{\mathrm{ac1}} \cos(\Omega t) \tag{4}$$

$$L_{21} \frac{di_2(t)}{dt} + R_{21} i_2(t) + \frac{1}{C_{21}} \int i_2(t) dt + C_c \int i_1(t) dt = 0 \tag{5}$$

where, comparing equations (4) and (5), the correspondence between equivalent mass-spring-damping and resistance-inductance-capacitance can be obtained as:

$$\begin{aligned} &L_{11} = \frac{m_1}{\eta_1}, \ R_{11} = \frac{c_{11}}{\eta_1}, \ C_{11} = \frac{\eta_1}{k_{11}}, \ C_c = \frac{\eta_1}{k_{\mathrm{c}}} \\ &L_{21} = \frac{m_2}{\eta_2}, \ R_{21} = \frac{c_{21}}{\eta_2}, \ C_{21} = \frac{\eta_2}{k_{21}} \end{aligned} \tag{6}$$

As shown in the Figure 4, the equivalent circuit model of two degrees of freedom with parasitic feedthrough effects is developed, in which the effective impedances of resonator 1 and resonator 2 as well as the coupling capacitor branch are as follows:

$$Z_1 = \frac{1}{\dfrac{1}{R_{11} + \dfrac{1}{j\Omega C_{11}} + j\Omega L_{11}} + j\Omega C_{\mathrm{f1}}} \tag{7}$$

$$Z_2 = R_{21} + \frac{1}{j\Omega C_{21}} + j\Omega L_{21} \tag{8}$$

$$Z_c = \frac{1}{j\Omega C_{\mathrm{c}}} \tag{9}$$

Z_1 is the impedance of the resonator 1 branch with the feedthrough capacitor C_{f1} connected in parallel, Z_2 is the impedance of resonator 2, and Z_c is the impedance of the coupling capacitor branch.

In the circuit shown in Figure 4, the expressions for the current response i_{s1} of resonator 1 and i_{s2} of resonator 2 are as follows:

$$i_{\mathrm{s1}} = V \times Y_1(\Omega) \tag{10}$$

$$i_{\mathrm{s2}} = i_{\mathrm{m1}} + i_{\mathrm{f2}} = V \times Y_2(\Omega) \tag{11}$$

where $Y_1(\Omega)$ and $Y_2(\Omega)$ are the conductances of the loop and the transfer function of the resonant electrical model of the parasitic signal, and the loop conductance is a function of the excitation frequency Ω, which can be expressed:

$$\begin{aligned} Y_1(\Omega) &= \frac{1}{Z_1 + \dfrac{1}{\dfrac{1}{Z_2} + \dfrac{1}{Z_{\mathrm{c}}}}} = \frac{1}{Z_1 + \dfrac{Z_2 Z_{\mathrm{c}}}{Z_2 + Z_{\mathrm{c}}}} \\ &= \frac{1}{\dfrac{1}{\dfrac{1}{R_{11} + \dfrac{1}{j\Omega C_{11}} + j\Omega L_{11}} + j\Omega C_{\mathrm{f1}}} + \dfrac{\left(R_{21} + \dfrac{1}{j\Omega C_{21}} + j\Omega L_{21}\right) j\Omega C_{\mathrm{c}}}{R_{21} + \dfrac{1}{j\Omega C_{21}} + j\Omega L_{21} + j\Omega C_{\mathrm{c}}}} \end{aligned} \tag{12}$$

To simplify the calculation, assuming $R_{11}=R_{21}=R_m$, $C_{11}=C_{21}=C_m$, $L_{11}=L_{21}=L_m$, the expression of admittance Y_1 (Ω) is obtained after simplification:

$$Y_1(\Omega)=\frac{\left(\frac{2r_2r_5}{R_m}+\frac{2r_2r_6}{R_m}+2C_cr_2r_3\Omega-C_{f1}r_1r_5\Omega-C_cr_1r_6\Omega-C_cC_m^2r_1R_m\Omega^2\right)}{r_1^2+4r_2^2}$$
$$+\frac{j\left(\frac{r_1r_5}{R_m}+\frac{r_1r_6}{R_m}+C_cr_1r_3\Omega+2C_{f1}r_2r_5\Omega+2C_cr_2r_6\Omega+2C_cC_m^2r_2R_m\Omega^2\right)}{r_1^2+4r_2^2}=G_1+jB_1$$
(13)

After obtaining the relationship between admittance $Y_1(\Omega)$, the expression for admittance $Y_2(\Omega)$ can be obtained based on the relationship between parallel circuits:

$$\begin{aligned}Y_2(\Omega)&=Y_1(\Omega)\times\frac{Z_c}{Z_2+Z_c}+j\Omega C_{f2}\\&=\frac{\frac{R_1r_5}{R_m\Omega}+C_cC_m^2R_mB_1\Omega}{C_m^2+C_cr_4+C_c^2C_m^2R_m^2\Omega^2}+j\left(\frac{\frac{r_5B_1}{R_m\Omega}-C_cC_m^2R_1R_m\Omega}{C_m^2+C_cr_4+C_c^2C_m^2R_m^2\Omega^2}-\Omega C_{f2}\right)\\&=G_2+jB_2\end{aligned} \quad (14)$$

Admittance $Y_1(\Omega)$ and $Y_2(\Omega)$ are both complex, where the real parts G_1 and G_2 are called conductivity, and the imaginary parts B_1 and B_2 are called admittance. The amplitude of complex admittance can be quantified as a function of frequency

$$|Y_1(\Omega)|=\sqrt{G_1^2+B_1^2},\quad |Y_2(\Omega)|=\sqrt{G_2^2+B_2^2} \quad (15)$$

The expressions for coefficients G_1, G_2, B_1 and B_2 are as follows:

$$\begin{cases}G_1=\frac{\left(\frac{2r_2r_5}{R_m}+\frac{2r_2r_6}{R_m}+2C_cr_2r_3\Omega-C_{f1}r_1r_5\Omega-C_cr_1r_6\Omega-C_cC_m^2r_1R_m\Omega^2\right)}{r_1^2+4r_2^2}\\B_1=\frac{\left(\frac{r_1r_5}{R_m}+\frac{r_1r_6}{R_m}+C_cr_1r_3\Omega+2C_{f1}r_2r_5\Omega+2C_cr_2r_6\Omega+2C_cC_m^2r_2R_m\Omega^2\right)}{r_1^2+4r_2^2}\\G_2=\frac{\frac{R_1r_5}{R_m\Omega}+C_cC_m^2R_mB_1\Omega}{C_m^2+C_cr_4+C_c^2C_m^2R_m^2\Omega^2}\\B_2=\left(\frac{\frac{r_5B_1}{R_m\Omega}-C_cC_m^2G_1R_m\Omega}{C_m^2+C_cr_4+C_c^2C_m^2R_m^2\Omega^2}-\Omega C_{f2}\right)\end{cases} \quad (16)$$

The expressions for parameters r_1, r_2, r_3, r_4 and r_5 are as follows:

$$\begin{cases} r_1 = r_3 + r_4 - C_c C_m^2 R_m^2 \Omega^2 \\ r_2 = r_4 + r_5 \\ r_3 = C_{f1} - 2C_{f1} C_m L_m \Omega^2 - C_{f1} C_m^2 R_m^2 \Omega^2 + C_{f1} C_m^2 L_m^2 \Omega^4 \\ r_4 = C_c + 2C_m - 2C_c C_m L_m \Omega^2 - 2C_m^2 L_m \Omega^2 + C_c C_m^2 L_m^2 \Omega^4 \\ r_5 = C_c C_m R_m \Omega - C_c C_m^2 L_m R_m \Omega^3 + C_m^2 R_m \Omega \\ r_6 = C_{f1} C_m R_m \Omega - C_{f1} C_m^2 R_m L_m \Omega^3 \end{cases} \tag{17}$$

According to equation (15), the amplitude frequency response of the coupled resonator at different feedthrough is shown in Figure 5.
